# Postbiotics in pediatric clinical practice: position paper from Special Group of Latin American Society of Pediatric Gastroenterology, Hepatology and Nutrition (LASPGHAN)

**DOI:** 10.3389/fnut.2025.1716791

**Published:** 2025-12-17

**Authors:** Rodrigo Vázquez-Frias, Gabriel Vinderola, Ana Teresa Abreu y Abreu, Diana Angulo, Natasha Giler-Párraga, Liliana Ladino, Carolina Ortiz, Sebastián Pereira, Juan Pablo Bustamante

**Affiliations:** 1 Latin American Society of Pediatric Gastroenterology, Hepatology and Nutrition, Mexico City, Mexico; 2 Subdirección de Gestión de la Investigación, Hospital Infantil de México Federico Gómez, Ciudad de México, Mexico; 3 Sociedad Mexicana de Microbiota, Ciudad de México, Mexico; 4 Instituto de Lactología Industrial (UNL-CONICET), Facultad de Ingeniería Química, Universidad Nacional del Litoral, Santa Fe, Argentina; 5 Hospital Nacional Docente Madre Niño “San Bartolomé,” Lima, Peru; 6 Universidad de las Américas, Quito, Ecuador; 7 Centro de Investigación y Educación en Nutrición CIENutrition, Bogotá, Colombia; 8 Gastro Care for Kids, Ciudad de Guatemala, Guatemala; 9 Hospital General Pediátrico Niños de Acosta Ñu, Hospital Central del Instituto de Previsión Social, Asunción, Paraguay; 10 Facultad de Ingeniería, Universidad Nacional de Entre Ríos, Oro Verde, Argentina; 11 Consejo Nacional de Investigaciones Científicas y Técnicas, CONICET, Buenos Aires, Argentina

**Keywords:** postbiotics, pediatric, clinical practice, microbiome, inactivated microorganisms

## Abstract

Postbiotics, defined by the ISAPP as preparations of inanimate microorganisms and/or their components that confer health benefits, represent a promising category of microbiome-derived solutions. This position paper highlights their clinical relevance, particularly in pediatrics, while addressing key aspects of definition, safety, quality, and strain-level specificity. Evidence supports the use of *Lactobacillus* LB –including *L. fermentum* CNCM I-2998 and *L. delbrueckii* subsp. lactis CNCM I-4831– in reducing the duration and severity of acute diarrhea in children. Other strains, such as *Bifidobacterium breve* C50, *Streptococcus thermophilus* 065, *Lacticaseibacillus paracasei* CBA L74, *Lactiplantibacillus plantarum* LPL28, and *Ligilactobacillus salivarius* AP-32, show promise in preventing infections, supporting oral health, and modulating immune responses. Additional postbiotics, including *Limosilactobacillus reuteri* DSM 17648, expand their potential into metabolic and gastrointestinal disorders. Collectively, postbiotics emerge as clinically valuable interventions, bridging science and medical practice.

## Introduction

The use of non-viable microorganisms capable of delivering health benefits–the counterpart to probiotics–is not new in clinical practice. However, unlike probiotics, a clear category for them has not yet been fully defined, understood, and communicated. This position paper does not aim to provide a systematic review of the health benefits of non-viable microbes. This work aims at adopting a definition for the term postbiotics, to communicate it to the pediatric community, to clarify key concepts and to highlight selected postbiotics currently available in LASPGHAN countries and abroad for application in pediatrics.

Guided by the definition of postbiotics provided by the International Scientific Association for Probiotics and Prebiotics (ISAPP definition), our objective is to provide an academic framework highlighting their clinical applications, the strain specificity, mechanisms of action, safety, and quality aspects. Evidence will be discussed for postbiotics including *Lactobacillus* LB (a heat-inactivated mixture of the strains *Limosilactobacillus fermentum* CNCM I-2998 and *Lactobacillus delbrueckii* subsp. lactis CNCM I-4831, and fermented culture medium), used to manage acute and chronic diarrhea, as well as antibiotic associated diarrhea, *Bifidobacterium breve* C50 and *Streptococcus thermophilus* 065, used in infant formulas or *Lacticaseibacillus paracasei* CBA L74, *Lactiplantibacillus plantarum* LPL28, and *Ligilactobacillus salivarius* AP-32, used prevention of common infections to atopic dermatitis and oral health. Additional postbiotics such as *Limosilactobacillus reuteri* DSM 17648 have shown promise in managing *H. pylori* infection and metabolic disorders, while inactivated *Lactobacillus* LB has also demonstrated benefits in treatment and improved eradication rates (1).

## Definition of postbiotics

**FIGURE 1:**
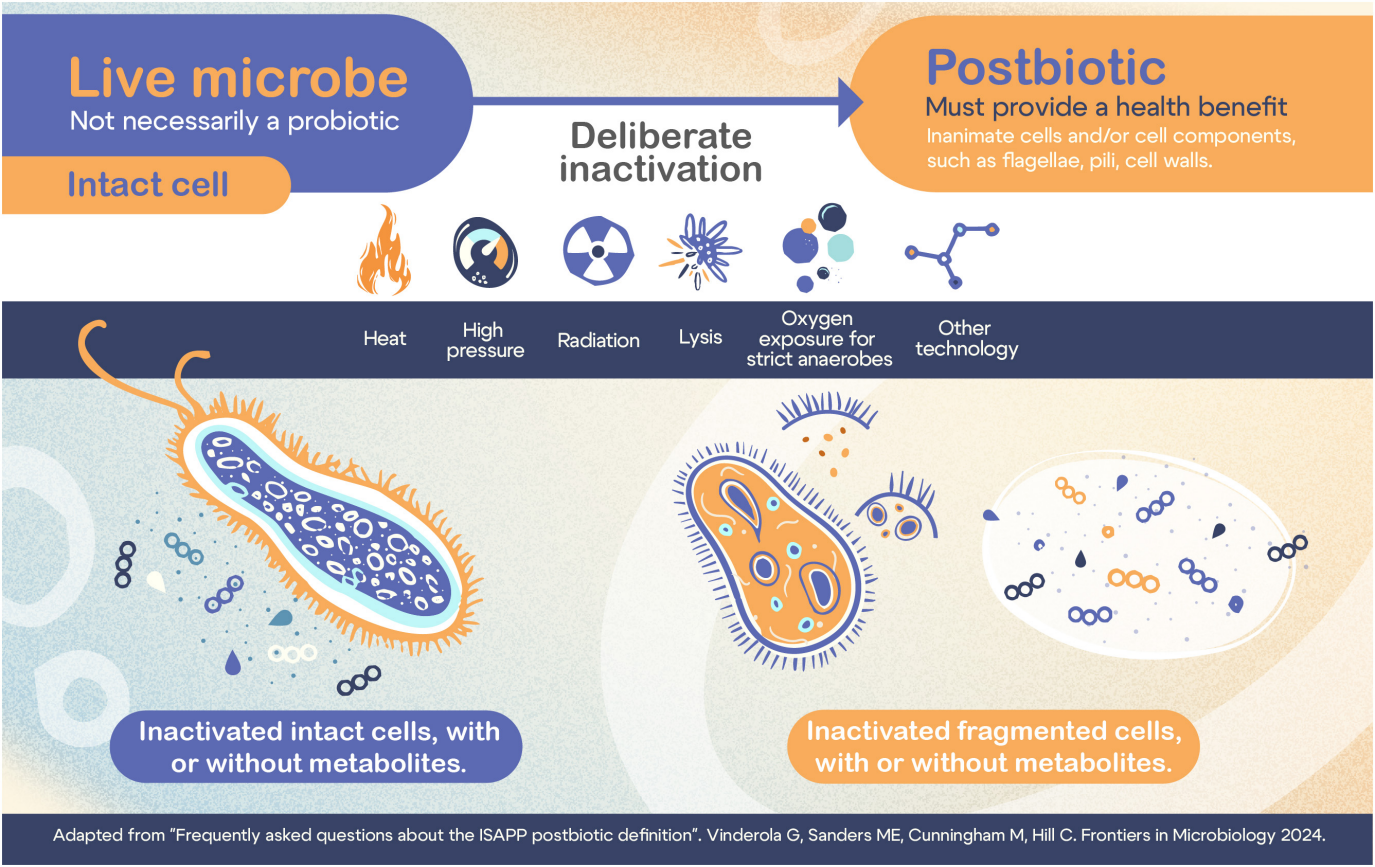
Schematic representation of the production of postbiotics, their main inactivation methods and key components.

The term postbiotics has been conceptualized in different ways in the last 15 years. For instance, it was used to refer to metabolites produced by the gut microbiota, beneficial metabolites produced by probiotics or inactivated microorganisms able to confer a health benefit (2). In addition, several definitions were proposed for this term, again conceptualizing it either as metabolites or as inactivated microorganisms (3). In this work, we adopt and support the definition of postbiotics proposed by the ISAPP, that stated that a postbiotic is a preparation of inanimate microorganisms and/or their cell components that confer a health benefit on the host (4). Figure 1 shows a schematic representation of the production of postbiotics, their main inactivation methods and key components.

The term postbiotics here encompasses all previous terms used to refer to the health properties delivered by non-viable microbes such as: non-viable probiotics, heat-inactivated probiotics, inactivated probiotics, heat-treated probiotics, heat-killed probiotics, tyndallized probiotics, postbiotics, paraprobiotics, ghost probiotics, cell lysates or cell fragments, being this list non-exhaustive and acknowledging that other terms may have been used too. The term preparation leads us to consider the technological process used for the life termination of the microorganisms, which is generally a heat treatment, but it could alternatively be radiation, lysis, high pressure, chemical inactivation or oxygen exposure for strict anaerobes such as *Akkermansia*, among others. The term inanimate refers to the lack of viability of the microorganisms, the lack of capacity of microbial cells to further replicate in the culture medium used for the growth of the progenitor strain. Inanimate can be also understood as inactivated, taking into consideration that inactivated does not mean inactive, or unable to confer a health benefit. Cells lacking the capacity to grow, to replicate, may still deliver health benefits by the mechanisms of action later described in this paper. The term components refer to structural cell components, components that make up the microbial cell (cell walls mainly), and not to cytoplasmatic or secreted metabolites. However, metabolites can be present, or not, in the preparation. In fact, many postbiotic products currently available in the market do include microbial metabolites, as cells are not extensively washed before the application of the inactivation step (5). The core of the ISAPP definition of postbiotics is the presence of inanimate microbial cells in the preparation, either as intact cells or as cell fragments (or cell components), the presence of metabolites in the preparation is not mandatory and it is up to the manufacturer, but they can significantly contribute to the health benefit.

## Mechanisms of action

There is still the misconception that life is a prerogative for a microbe to confer a health benefit, and then it has been largely underscored the capacity of non-viable cells to be used as health-delivery agents. In fact, many probiotic products may contain significant amounts of dead cells, derived from the technological process of freeze-drying used for probiotic product preparation. This fraction of dead cells may significantly contribute to the health benefit ascribed to probiotics (6), though it should not be considered postbiotics. Health benefits of postbiotic preparations may be delivered by the inanimate cells, by their cell components or by the microbial metabolites present in the preparation, or by a combination of all these fractions. Postbiotics are generally complex preparations that may encompass different bioactive compounds with multiple mechanisms of action and in most cases the exact mechanisms of action by which postbiotics exert their benefit and their role in human health were not clearly clarified. In many cases, these mechanisms may be shared with probiotics. In the gut, postbiotics may exert their benefits by the modulation of the gut-associated immune system, by enhancement of epithelial barrier functions, by the modulation of the gut microbiome, by modulation of systemic metabolic responses, by systemic signaling via the nervous system (gut-brain axis) or by a combination of some or all of these mechanisms (4, 7). Mechanisms of action are strain-dependent and cannot be generalized to the whole category of postbiotics. A full mechanism elucidation for each postbiotic available is not a requisite for efficacy. As it happens for probiotics, in most cases the exact way that a postbiotic, or a probiotic, exerts its health benefits is not completely understood. In some cases, evidence obtained from simplified models such as cell lines, organoids culture or animal assay, may shed light and allow to hypothesize the mechanisms underlying the health benefit of a postbiotic. One may reasonably think that mechanisms of action linked to cell viability will not be exerted by postbiotics, as would be the case of lactose intolerance symptom relief, for which the active version of the enzyme lactase is needed. This would be the case if the postbiotic was produced by heat inactivation. However, if the postbiotic was produced by technological processes that preserves enzymatic activity (cell disruption, radiation, lysis), lactase activity may still be expected. Then, the type of bioactive components present in a postbiotic preparation will be necessarily linked to the technological process used for life termination. Still it is not reasonable to expect health benefits that are intimately linked to the metabolic activity of intact viable cells.

In particular, microbial components like lipoteichoic acids and peptidoglycans can stimulate pattern recognition receptors (PRRs), such as Toll-like receptors, enhancing innate and adaptive immunity (8, 9). Metabolites present in the postbiotic preparation like butyrate and indole derivatives support tight junction integrity and mucin production (4) or the growth of bifidobacteria (10). Other mechanisms of action may be mediated by bacteriocins and organic acids that lowers gut pH and inhibits pathogen colonization (10, 11) or by the inhibition of NF-κB pathways and cytokine modulation reduce local and systemic inflammation (12).

## Similarities and differences between prebiotics, probiotics, synbiotics, postbiotics and fermented foods

The so-called “biotics” family comprises diverse but interconnected concepts, all aimed at delivering specific health benefits. Although they share this ultimate goal, their definitions, applications, and clinical implications differ. This section is accompanied by a comparative table that summarizes their consensus definition, whether they involve live microbes or not, some examples, and key considerations, offering a practical framework for distinguishing their roles in healthcare. For completeness, fermented foods are also included in this comparison, as they are often confused with probiotics and postbiotics. While fermented foods may contain live and non-viable microorganisms or microbial metabolites, they are not classified as “biotics” unless they meet the strict consensus definitions. Similarities and differences between prebiotics, probiotics, synbiotics, postbiotics and fermented foods according to ISAPP definitions are summarized at Table 1.

**TABLE 1:** Similarities and differences between prebiotics, probiotics, synbiotics, postbiotics and fermented foods according to ISAPP definitions.

Term	Consensus definition	Simple concept	Live microbes present?	Health benefit required?	Examples	Key considerations	References
Prebiotic	A substrate that is selectively utilized by host microorganisms conferring a health benefit on the host	Food for beneficial microbes residing on or within the host	No	Yes	Inulin, FOS, GOS, resistant starch, HiMO, lactulose	Look for appropriate characterization and research to confirm the prebiotic status of an ingredient.	Gibson et al. (13)
Probiotic	Live microorganisms that, when administered in adequate amounts, confer a health benefit on the host	Live microbes that are good for your health	Yes	Yes	*Bifidobacterium animalis* subsp. lactis XYZ, *L. plantarum* ABC	Identity must be confirmed through genome sequencing at strain level. Sufficient viability must be preserved through to end of shelf life.	Hill et al. (14)
Synbiotic	A mixture comprising live microorganisms and substrate(s) selectively utilized by host microorganisms that confers a health benefit on the host	Complementary synbiotic: is a mixture of probiotic + prebiotic. Synergistic synbiotic: contains a live microbe and a substrate that it can use for growth.	Yes	Yes	Complementary: inulin + *B. animalis* subsp. lactis XYZ Synergistic: co-selected live microbe + substrate Products with added probiotics (strain identified level) and fiber-rich ingredients (such as some infant formulas, some yogurts)	A health benefit must be shown for a synbiotic as combined, not just the probiotic alone and the prebiotic alone.	Swanson et al. (15)
Postbiotic	Preparation of inanimate microorganisms and/or their components that confers a health benefit on the host	Non-viable microbes and/or cell components with or without metabolites	No. Live microbes are the starting point to make a postbiotic, but they are intentionally inanimated	Yes	Certain infant formulas, bacterial lysates, inactivated yeast fermentates, and inactivated bacteria. Human milk is a source of putative postbiotic-like components.	Requires deliberate inactivation of microbes. Identity must be confirmed at strain level. Purified metabolites alone do not qualify (e.g., butyric acid).	Salminen et al. (4)
Fermented Foods	Foods made through desired microbial growth and enzymatic conversions of food components	Foods that are made through the growth of live microbes	Sometimes (not required in final product)	No	Yogurt, kefir, sauerkraut, kombucha, sourdough bread	Microbes present not required to be defined and may be live or dead.	Marco et al. (16)

## Safety aspects

In principle, postbiotics may be regarded as safer than probiotics for certain applications, especially in pediatric populations, neonates, and immunocompromised patients. However, safety must be demonstrated on a strain-specific basis. Postbiotics minimize the risks of translocation, bacteremia, and sepsis associated with the administration of live microorganisms (17, 18). Adding postbiotics to infant formula is considered safe, as evidence shows no increase in serious adverse events, colic, diarrhea, vomiting, or other gastrointestinal disorders. Also growth parameters such as weight, length, and head circumference were unaffected. Strains used include heat-treated *Bifidobacterium breve* C50 and *Streptococcus thermophilus* 065, or *Lacticaseibacillus paracasei* CBA L74 (19). Overall, postbiotics provide a safe option in infant formula, without harmful effects on infant health and development (20).

Heat-killed *Lactobacillus* LB has demonstrated safety across numerous clinical trials, with no adverse events reported in neonates, infants, or children with acute illness (21, 22). Clinical trials in pediatrics evaluating postbiotics, particularly heat-killed *Lactobacillus* LB, in acute diarrheal disease show a favorable safety profile. Across multiple RCTs in diverse settings, no serious adverse effects were reported, and tolerability was comparable to placebo or standard therapy. Safety assessments indicated that children, including infants, tolerated postbiotic supplementation well, with no evidence of increased complications or treatment discontinuations. The main reported events were mild, self-limiting gastrointestinal symptoms, indistinguishable from background illness. These findings suggest that postbiotics are safe in pediatric populations with acute diarrhea, supporting their use as adjuncts to rehydration therapy, although efficacy outcomes remain heterogeneous (23).

## Are there postbiotic strains?

A critical point in advancing the field of postbiotics is the recognition that they must be identified at the strain level, just as probiotics are. The rationale is straightforward: biological activity is not uniform across species, or even across subspecies, but rather determined by the unique molecular repertoire of each strain. Once a microorganism is inanimated, its viability may be lost, but its strain-specific structural components and metabolites remain, continuing to shape its physiological and clinical effects. In this sense, the concept of postbiotic strains is particularly important in clinical practice, as it underscores the relevance of strain-specific effects. *Lactobacillus* LB is one such example, whose heat-killed cells and metabolites remain biologically active and have consistently demonstrated favorable outcomes, such as shorter duration of diarrhea, in several randomized controlled trials (21, 22). This confirms that the therapeutic value of this mixture of 2 strains is preserved in the absence of viability, in its post-inanimated form. Similarly, *Bifidobacterium breve* C50 and *Streptococcus thermophilus* 065, when used to ferment infant formulas, retain their distinct strain-specific signatures. Clinical studies show that these formulas are not only safe and well tolerated but also reduce the severity of diarrheal episodes and support immune and gut maturation, benefits directly tied to the characteristics of these strains (8, 24).

## Quality aspects of postbiotics

Ensuring quality in postbiotic formulations requires strict control of manufacturing processes. Standardization of inactivation methods is essential to guarantee both safety and efficacy reproducibility. Measurement of microbial content should move from traditional colony-forming units (CFUs) to other tools able to quantify non-viable cells such as flow cytometry or quantitative PCR, among other potential analytical tools. Equally important are strain purity and accurate labeling, ensuring consistency and traceability. Future directions may include the quantification of key components of the postbiotic preparation (5). This step would provide stronger links between product composition and clinical outcomes. Finally, regulatory frameworks, currently treating postbiotics either as food supplements or medicines, are expected to evolve, providing clearer criteria to enhance consumer safety and product transparency.

## Clinical utility of postbiotics

We aimed at briefly examining the gastrointestinal and nutritional health benefits of postbiotics, while also acknowledging additional areas of application in pediatric populations.

### In healthy children

Although there are studies evaluating postbiotics in healthy children, there is currently no recommendation to administer biotics, neither probiotics nor postbiotics, to healthy pediatric populations in the absence of specific medical conditions.

### In acute pediatric diarrheal disease

There are no universally accepted definitions or criteria for characterizing or managing bacterial versus viral diarrhea. Divergences exist in defining stool consistency and frequency thresholds. The standard treatment for diarrhea remains centered on oral rehydration therapy with low-osmolarity solutions, along with nutritional follow-up that emphasizes both prevention and management. This includes, when available, the continuation of breastfeeding as an early refeeding strategy to help preserve intestinal trophism. In this context, probiotics and postbiotics serve as adjunctive therapies. Clinical evidence supports the use of heat-killed *Lactobacillus* LB for the reduction of the duration and severity of acute diarrhea (21, 25), which includes a combination of *L. fermentum* CNCM I-2998 and *L. delbrueckii* subsp. lactis CNCM I-4831.

In a randomized, double-blind, placebo- and reference-controlled trial, 103 infants and toddlers with acute diarrhea were enrolled, with 38 receiving heat-killed *Lactobacillus* LB, 33 receiving placebo, and 32 receiving loperamide. All children received standard WHO-based therapy with rehydration and diet. Results showed that the mean time to first normal stool was significantly shorter in the *Lactobacillus* LB group (approximately 41 h with oral rehydration, 49.7 h overall) compared with placebo (67.8 h with oral rehydration, 64.7 h overall; *p* ≤ 0.05). This effect was due to a shorter stool-free interval before recovery. Recovery rates were high and similar across groups (≈85%–90%). No serious adverse events were observed, and tolerability was excellent (25).

In another randomized, double-blind, placebo-controlled trial included 73 Thai children with acute watery diarrhea, of whom 37 received heat-killed *Lactobacillus* LB and 36 received placebo, both alongside oral rehydration therapy. The study found that the mean duration of diarrhea was significantly shorter in the heat-killed *Lactobacillus* LB group (1.8 ± 1.1 days) compared with the placebo group (2.4 ± 1.5 days; *p* < 0.05). Treated children also required fewer unscheduled intravenous rehydrations. No serious adverse events occurred, and the intervention was well tolerated (21).

In a randomized, double-blind, placebo-controlled trial, 80 hospitalized Ecuadorian children with acute diarrhea, randomized to receive heat-killed *Lactobacillus* LB (*n* = 42) or placebo (*n* = 38), in addition to standard oral rehydration. The results demonstrated that the duration of diarrhea was significantly shorter in the heat-killed *Lactobacillus* LB group (1.6 days) compared with placebo (2.6 days; *p* < 0.01). No serious adverse events were reported, and tolerability was excellent (26).

Another randomized, double-blind, placebo-controlled trial included 80 Peruvian children with acute watery diarrhea, assigned to receive heat-killed *Lactobacillus* LB (*n* = 40) or placebo (*n* = 40), alongside oral rehydration therapy. The results showed that the median duration of diarrhea was significantly shorter in the heat-killed *Lactobacillus* LB group (0.3 days) compared with placebo (1.3 days; *p* < 0.01). No serious adverse events occurred, and treatment was well tolerated (22, 27).

### In other pediatric gastrointestinal diseases

Postbiotics may promote mucosal healing, and reduce inflammation in functional gastrointestinal disorders and inflammatory bowel disease. They could also play a role in the management of patients with bacterial overgrowth syndrome (28). Further studies in the pediatric population are needed to establish recommendations. They may also be considered for adjunctive use in the management of *Helicobacter pylori*, as suggested by studies with inactivated *Lactobacillus* LB in treatment and improved eradication rates (1), *Limosilactobacillus reuteri* (*Lactobacillus reuteri* in the previous classification) DSM 17648 in adult patients (29). However, these findings are limited to adults, and well-designed pediatric trials are still needed to confirm efficacy and safety in children.

### In other pediatric conditions

Infant formulas with postbiotics obtained from milk fermented with *Bifidobacterium breve* C50 and *Streptococcus thermophilus* O65 -and their metabolites-, including the oligosaccharide 3’-GL, have demonstrated to be safe and to contribute to the development of the gut microbiota and the gut associated immune system and for the management of infant colics (30). The infant formula containing the postbiotic *Bifidobacterium lactis* BPL1 demonstrated safety and tolerability (31) and impact on the gut microbiota of healthy children (32).

Emerging evidence suggests potential roles in reduction of body fat, obesity, respiratory tract infections, eczema, and oral health, primarily through systemic immune modulation and microbiome crosstalk. Childhood obesity is a major health concern linked to metabolic disorders. Postbiotics, due to their safety and stability, are emerging as promising interventions. The use of postbiotics in dermatology is supported by a growing body of evidence from both preclinical and clinical studies, which have demonstrated their potential in treating a range of skin conditions from acne to eczema. Further, Torii et al. demonstrated that oral administration of *Lactobacillus acidophilus* L-92 significantly improved atopic dermatitis in Japanese children by regulating the T helper type-1/T helper type-2 (Th1/Th2) immune axis (33).

Although direct clinical trials in IBD patients are still lacking, postbiotics appear to be a promising alternative: they have shown anti-inflammatory, immunomodulatory, antioxidant, and mucosal-protective effects in preclinical studies, which could contribute to the prevention and management of IBD (34). However, clinical evidence in children is still limited.

A multicenter, randomized, double-blind, placebo-controlled trial in healthy children (12–48 months) evaluated a fermented formula containing *L. paracasei* CBA L74 (*n* = 66) versus placebo (*n* = 60) during the winter season. Over 3 months, children consuming the fermented formula experienced fewer episodes of common infectious diseases (CIDs), including acute gastroenteritis and respiratory infections, alongside significant increases in innate (α- and β-defensins, cathelicidin) and adaptive (secretory IgA) immune markers. These findings support the potential of postbiotics as an effective preventive strategy in pediatric populations, although further evidence is still required (35).

In addition, *L. paracasei* CBA L74, *L. plantarum* LPL28, *L. salivarius* AP-32 and *S. thermophilus* 065 demonstrate anticariogenic activity by inhibiting *Streptococcus mutans* growth, reducing biofilm formation, and modulating virulence. Clinical trials in children and adults have reported reduced salivary *S. mutans*, improved oral pH stability, enhanced secretory IgA, and better oral health outcomes (36, 37). These results highlight the dual potential of postbiotic strains in promoting both gut health and oral health through barrier support and immune modulation.

## Conclusion

Postbiotics, defined as preparations of inanimate microorganisms and/or their components that confer health benefits, represent a promising and growing category in pediatric care. Postbiotic products in Latin American countries and abroad available for managing different pediatric conditions include heat-killed *Lactobacillus* LB (*L. fermentum* CNCM I-2998 and *L. delbrueckii* subsp. lactis CNCM I-4831, and fermented culture medium), infant formula produced with *Bifidobacterium breve* C50 and *Streptococcus thermophilus* 065, *Lacticaseibacillus paracasei* CBA L74 or *Bifidobacterium lactis* BPL1, among others. Their safety, stability, and strain-specific activity underscore their clinical utility, especially in vulnerable populations. Standardization of definitions, manufacturing quality, and regulatory clarity remain essential to fully integrate postbiotics into evidence-based practice and advance their therapeutic applications. This position paper invites healthcare professionals of the pediatric community to conceptualize postbiotics under the perspective of the LASPGHAN with the ISAPP definition.

## Data Availability

The original contributions presented in this study are included in this article/supplementary material, further inquiries can be directed to the corresponding authors.
